# Lack of correlation between cerebral arteriovenous malformation angioarchitectural and hemodynamic characteristics and systemic inflammation

**DOI:** 10.1007/s00701-025-06464-0

**Published:** 2025-04-15

**Authors:** Tatiana Abou-Mrad, Laura Stone McGuire, Jessica Hossa, Peter Theiss, Mpuekela Tshibangu, Adrusht Madapoosi, Fady T. Charbel, Ali Alaraj

**Affiliations:** Department of Neurosurgery, University of Illinois Chicago, 912 S. Wood St 451N - MC 799, Chicago, IL 60612 USA

**Keywords:** Angioarchitecture, Cerebral arteriovenous malformation, Flow, Hemodynamics, Inflammation, Systemic inflammatory index

## Abstract

**Purpose:**

Plasma-based inflammatory biomarkers have gained attention in cerebrovascular pathologies, with studies suggesting links to high-risk features. This study investigates the association between systemic inflammatory markers and cerebral arteriovenous malformation (AVM) angioarchitectural and hemodynamic characteristics.

**Methods:**

A single-center database of AVM patients (2007–2023) was queried. Patients with unruptured, supratentorial AVMs, baseline quantitative magnetic resonance angiography, and complete blood counts at admission were included. Biomarkers analyzed included white blood cell (WBC) count, absolute neutrophil count (ANC), absolute monocyte count (AMC), absolute lymphocyte count (ALC), and platelet count. Neutrophil–lymphocyte ratio (NLR), lymphocyte-monocyte ratio (LMR), platelet-lymphocyte ratio (PLR), and systemic inflammation index (SII) were calculated. AVM characteristics and hemodynamic properties were assessed.

**Results:**

86 patients met inclusion criteria. No significant correlations were found between systemic inflammatory markers and AVM size, morphology, venous stenosis, or Spetzler-Martin grade. While WBC count and ANC weakly correlated with flow index (*p* < 0.05), AVM flow showed no consistent associations with inflammatory markers.

**Conclusion:**

Systemic inflammatory markers do not consistently correlate with unruptured AVM angioarchitecture or hemodynamics. These findings suggest systemic inflammation may have limited relevance to sporadic AVM pathology. Future studies should explore localized inflammatory biomarkers to better understand AVM behavior.

## Introduction

Cerebral arteriovenous malformations (AVMs) are complex vascular lesions that pose significant clinical challenges due to their propensity for rupture, often resulting in intracranial hemorrhage (ICH) with high associated morbidity and mortality. These anomalies, defined by direct arteriovenous shunting without intervening capillaries, exhibit an incidence of 0.94 to 1.34 per 100,000 person-years and typically present between the ages of 30 and 35 [23, 38]. The pathophysiological model implicates the absence of capillary beds, resulting in high-pressure blood flow through the AVM nidus with subsequent transmission to the venous system, which leads to venous hypertension and rupture [24]. Untreated AVMs carry an annual hemorrhage risk of 3–5%, which can reach as high as 34% in the presence of additional risk factors, such as prior hemorrhage, hypertension, venous stenosis, and deep venous drainage [1, 16, 38].

Emerging evidence highlights the role of inflammation in AVM pathophysiology, particularly in vascular dysmorphogenesis and rupture predisposition [27, 37]. Inflammatory processes, driven by genetic and molecular factors, may destabilize AVM vessel walls, compromising their structural integrity [18]. Systemic inflammatory biomarkers, such as the systemic immune-inflammation index (SII), have shown promise in evaluating inflammatory states across various cerebrovascular conditions [7]. The SII, which integrates peripheral lymphocyte, neutrophil, and platelet counts, was originally proposed in a variety of oncological research as a predictor of high risk of cancer recurrence or death [2]. Recently, the SII has demonstrated utility in various medical contexts, including ischemic stroke and intracranial aneurysms, where it correlates with disease severity and vessel instability, respectively [11, 14, 17, 31, 34]. Given these insights, this study explores the association between systemic inflammatory markers, particularly the SII, and AVM angioarchitectural and hemodynamic characteristics. The objective is to investigate whether systemic inflammation correlates with features that predispose AVMs to instability or rupture, or vice versa, thereby providing potential insights into AVM risk stratification and management.

## Materials and methods

### Human ethics and consent to participate

The study complied with institutional guidelines and was approved by the Institutional Review Board (IRB 2016–0659). Informed consent for diagnostic angiography was obtained from all patients, with stringent measures to ensure privacy and confidentiality.

### Study design and patient selection

This single-center, retrospective study utilized prospectively collected data from an institutional database comprising 647 patients with cerebral AVMs. Patients were included if they had unruptured, supratentorial lesions with baseline quantitative magnetic resonance angiography (QMRA), and a complete blood count (CBC) upon admission prior to any AVM treatment. Patients presenting with active bleeding from AVM rupture or any type of hemorrhage were excluded to minimize the impact of acute inflammation caused by recent hemorrhage. Moreover, patients with active infections (e.g., pneumonia, sepsis) were excluded from this study to minimize acute inflammatory confounding.

### Variables

#### AVM characteristics

Angioarchitectural data included AVM size, volume, nidus morphology (compact vs. diffuse), Spetzler-Martin grade, and the presence of feeder artery aneurysms, intranidal aneurysms, or venous stenosis (defined as more than 50% stenosis in one of the draining veins). Median values were used to categorize AVM size and volume. AVM volume was measured in the standard ellipsoid formula (A x B x C / 2). Nidus compactness was defined as tightly packed venous loops, while a diffuse nidus exhibited dispersed anomalous vessels within normal brain parenchyma. Two trained endovascular neurosurgeons assessed the angioarchitectural features using endovascular reports.

#### Inflammatory markers

CBC data were collected within 24 h after admission, and before any AVM treatment (including embolization, surgery, or radiosurgery). It included total white blood cell (WBC), absolute neutrophil count (ANC), absolute monocyte count (AMC), absolute lymphocyte count (ALC), and platelet count. Derived inflammatory indices included the neutrophil–lymphocyte ratio (NLR), lymphocyte-monocyte ratio (LMR), platelet-lymphocyte ratio (PLR), and systemic inflammation index (SII) were calculated: NLR divides the ANC by the ALC, LMR divides the ALC by the AMC, PLR divides the platelet count by ALC, and SII multiplies platelet count and ANC then divides by ALC. These measures have been used and validated in previous studies [2, 10].

#### Blood flow measurements

Blood flow measurements were attained with QMRA using the commercially available NOVA software (Non-invasive Optimal Vessel Analysis, VasSol, Inc., River Forest, Illinois). This technique has been previously validated and described in numerous studies involving cerebral vascular pathologies [3, 9, 13, 19, 20, 28, 29, 32, 33, 35]. Initial QMRA, prior to treatment, were used to calculate total AVM flow for this analysis. Total AVM flow was measured from a single draining vein when possible. Alternatively, total AVM flow was derived by taking the difference between the sum of flow in the primary arterial feeders relative to their contralateral counterparts:

$$\begin{array}{c}\left(ipsilateral A2 segment+middle cerebral artery+posterior cerebral artery\right)-\\ (contralateral A2 segment+middle cerebral artery+posterior cerebral artery\end{array}$$

The flow index was calculated as total AVM flow divided by volume.

### Statistical analysis

Data were assessed for normality before analysis. Relationships between continuous variables were evaluated using Pearson correlation, with partial Pearson correlation applied when controlling for additional covariates. All statistical analyses were performed using SPSS version 29 (IBM, Inc., Armonk, New York), with two-sided tests and a significance threshold of α = 0.05.

## Results

### Patient demographics

**Fig. 1:**
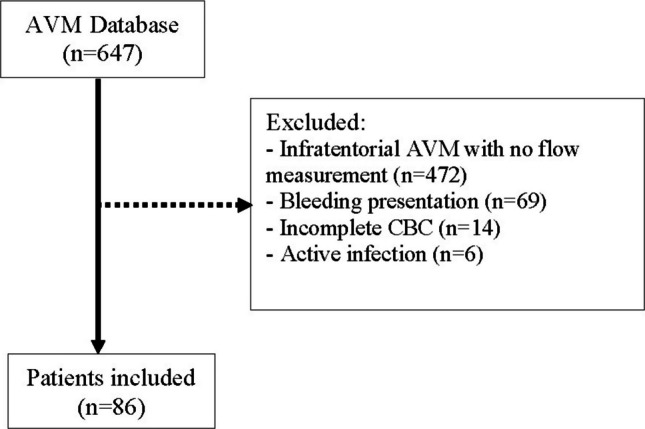
Flowchart outlining the patient selection process

A total of 86 patients met the inclusion criteria, with the selection process depicted in Fig. 1. The mean age of the cohort was 38.7 ± 15.0 years. Male patients constituted the majority (*n* = 48, 55.8%), while 38 patients (44.2%) were female. In terms of race and ethnicity, the distribution was as follows: 39.5% (*n* = 34) were White/Caucasian, 20.9% (*n* = 18) Black/African-American, 18.6% (*n* = 16) Latino, 2.3% (*n* = 2) Asian, and the remaining 18.6% (*n* = 16) identified as other. Regarding AVM characteristics, 14.0% (*n* = 12) of patients had feeder artery aneurysms, while 11.6% (*n* = 10) exhibited intranidal aneurysms. Venous stenosis was present in 52.3% (*n* = 45) of cases. Nidus morphology was classified as compact in 60.5% (*n* = 52) of patients and diffuse in 39.5% (*n* = 34). Table 1 summarizes the demographic and angioarchitectural characteristics of the cohort.

**Table 1:** Characteristics of AVM cohort

Factor	Mean ± SD or N (%)
Age	38.7 ± 15.0
Sex	
Female	38 (44.2%)
Male	48 (55.8%)
Race	
Black / African-American	18 (20.9%)
Latino	16 (18.6%)
Asian	2 (2.3%)
White / Caucasian	34 (39.5%)
Other	16 (18.6%)
Spetzler-Martin grade	
1	15 (17.4%)
2	29 (33.7%)
3	30 (34.9%)
4	7 (8.1%)
5	5 (5.8%)
Size (cm)	3.3 ± 1.4
< 4	52 (60.5%)
≥ 4	34 (39.5%)
Volume (mL)	14.4 ± 19.2
< 5.6	35 (40.7%)
≥ 5.6	51 (59.3%)
Feeder artery aneurysm	12 (14.0%)
Intranidal aneurysm	10 (11.6%)
Nidus	
Compact	52 (60.5%)
Diffuse	34 (39.5%)
Venous stenosis	45 (52.3%)

### AVM angioarchitectural characteristics

Analysis revealed limited significant associations between AVM angioarchitectural features and inflammatory markers (Table 2). LMR was significantly higher in females compared to males (*p* = 0.010). Patients with venous stenosis demonstrated a lower AMC (*p* = 0.021). Age showed a weak negative correlation with LMR (-0.254, *p* = 0.020). Other angioarchitectural features, including the presence of feeder aneurysms, intranidal aneurysms, venous stenosis, AVM size, volume, compactness, and grade, were not significantly associated with inflammatory markers.

**Table 2:** Association between AVM characteristics and inflammatory markers. *p*-value for each analysis listed in parentheses

Factor	WBC Total	ANC	AMC	ALC	Plt	NLR	LMR	PLR	SII
Age	-0.072 (0.505)	-0.057 (0.608)	0.099 (0.370)	-0.154 (0.163)	-0.118 (0.276)	0.125 (0.256)	-0.254 (0.020)*	0.128 (0.250)	0.065 (0.561)
Sex									
Male	8.3 ± 2.9	5.8 ± 2.9	0.5 ± 0.2	1.8 ± 0.7	224 ± 75	4.4 ± 4.1	4.0 ± 1.7	156 ± 108	987 ± 937
Female	7.6 ± 3.0 (0.288)	5.1 ± 2.9 (0.274)	0.5 ± 0.3 (0.698)	2.0 ± 0.9 (0.239)	235 ± 74 (0.474)	3.9 ± 4.1 (0.546)	5.3 ± 2.7 (0.010)*	160 ± 148 (0.875)	870 ± 900 (0.565)
Feeder aneurysm									
Yes	7.7 ± 2.8	5.4 ± 3.0	0.5 ± 0.4	1.7 ± 1.1	239 ± 49	5.9 ± 5.7	4.7 ± 2.4	279 ± 287	1454 ± 1201
No	8.0 ± 3.0 (0.748)	5.5 ± 3.0 (0.962)	0.5 ± 0.3 (0.954)	1.9 ± 0.8 (0.531)	228 ± 77 (0.650)	3.9 ± 3.7 (0.271)	4.6 ± 2.3 (0.916)	141 ± 75 (0.162)	862 ± 856 (0.162)
Intranidal aneurysm									
Yes	7.1 ± 2.3	4.4 ± 2.3	0.4 ± 0.3	2.1 ± 0.4	262 ± 134	2.3 ± 1.5	6.4 ± 3.3	124 ± 40	604 ± 381
No	8.1 ± 3.0 (0.441)	5.5 ± 2.3 (0.381)	0.5 ± 0.3 (0.578)	1.9 ± 0.9 (0.259)	227 ± 70 (0.306)	4.3 ± 4.2 (0.245)	4.5 ± 2.2 (0.053)	160 ± 130 (0.540)	954 ± 938 (0.410)
Size									
< 4 cm	8.5 ± 2.8	6.1 ± 2.9	0.5 ± 0.2	1.9 ± 0.9	229 ± 77	4.6 ± 4.3	4.7 ± 2.3	156 ± 113	1000 ± 861
≥ 4 cm	7.5 ± 3.0 (0.113)	4.9 ± 2.9 (0.056)	0.5 ± 0.3 (0.435)	1.9 ± 0.8 (0.896)	229 ± 71 (0.990)	3.7 ± 3.8 (0.295)	4.5 ± 2.4 (0.797)	159 ± 140 (0.921)	867 ± 973 (0.519)
Volume									
< 5.6 mL	8.4 ± 2.8	6.0 ± 3.1	0.5 ± 0.3	1.9 ± 0.9	215 ± 58	4.6 ± 4.5	4.6 ± 2.5	141 ± 80	934 ± 874
≥ 5.6 mL	7.7 ± 3.0 (0.236)	5.1 ± 2.8 (0.207)	0.5 ± 0.3 (0.844)	1.9 ± 0.8 (0.997)	239 ± 83 (0.126)	3.9 ± 3.7 (0.420)	4.6 ± 2.2 (1.000)	168 ± 145 (0.344)	933 ± 952 (0.996)
Venous stenosis?									
Yes	7.5 ± 2.6	5.1 ± 2.6	0.4 ± 0.3	1.9 ± 0.9	223 ± 76	4.3 ± 4.6	5.0 ± 2.0	168 ± 163	871 ± 847
No	8.6 ± 3.2 (0.075)	5.9 ± 3.3 (0.181)	0.6 ± 0.3 (0.021)*	1.9 ± 0.8 (0.857)	238 ± 73 (0.352)	4.0 ± 3.5 (0.740)	4.1 ± 2.5 (0.070)	148 ± 73 (0.485)	1016 ± 994 (0.480)
Nidus									
Diffuse	7.4 ± 2.9	4.9 ± 2.6	0.5 ± 0.3	1.9 ± 0.8	232 ± 78	3.6 ± 3.5	4.7 ± 2.4	174 ± 175	865 ± 888
Compact	8.4 ± 2.9 (0.119)	5.8 ± 3.1 (0.143)	0.5 ± 0.2 (0.306)	1.9 ± 0.9 (0.915)	227 ± 73 (0.755)	4.5 ± 4.4 (0.302)	4.6 ± 2.3 (0.899)	146 ± 82 (0.337)	976 ± 950 (0.596)
SM grade									
1	8.1 ± 1.9	5.5 ± 1.8	0.5 ± 0.2	1.9 ± 0.8	239 ± 49	3.8 ± 2.9	4.9 ± 2.1	154 ± 81	916 ± 745
2	8.3 ± 3.3	5.7 ± 3.3	0.5 ± 0.2	1.9 ± 0.8	222 ± 86	4.8 ± 5.2	4 4 ± 2.6	164 ± 163	988 ± 1021
3	8.0 ± 3.1	5.4 ± 3.3	0.5 ± 0.2	2.0 ± 0.8	235 ± 71	3.8 ± 3.7	4.7 ± 2.4	157 ± 121	959 ± 997
4	6.0 ± 1.3	3.4 ± 0.9	0.6 ± 0.4	1.8 ± 0.7	218 ± 74	2.1 ± 1.0	3.5 ± 1.7	128 ± 59	473 ± 326
5	8.1 ± 2.9 (0.631)	5.8 ± 2.2 (0.592)	0.4 ± 0.2 (0.560)	1.7 ± 1.2 (0.974)	217 ± 99 (0.913)	5.2 ± 3.3 (0.618)	5.0 ± 1.8 (0.805)	165 ± 102 (0.986)	971 ± 709 (0.850)

*WBC;* White blood cell, *ANC;* Absolute neutrophil count, *AMC;* Absolute monocyte count, *ALC;* Absolute lymphocyte count, *Plt;* Platelet, *NLR;* Neutrophil lymphocyte ratio, *LMR;* Lymphocyte monocyte ratio, *PLR;* Platelet lymphocyte ratio, *SII;* Systemic inflammatory index

*significance for* p *< 0.05

### AVMs hemodynamic properties

No consistent relationships were observed between hemodynamic properties and inflammatory biomarkers. While the flow index positively correlated with WBC count (0.238, *p* = 0.026) and ANC (0.285, *p* = 0.009), AVM flow showed no significant associations, suggesting that systemic inflammation is not a dominant factor in AVM pathology (Table 3). This also might suggest that fast arteriovenous shunting does not contribute to systemic inflammatory response.

**Table 3:** Association between AVM flow and inflammatory markers. p-value for each analysis listed in parentheses

Factor	WBC Total	ANC	AMC	ALC	Plt	NLR	LMR	PLR	SII
AVM flow	-0.025 (0.817)	-0.001 (0.995)	-0.056 (0.612)	0.004 (0.972)	0.162 (0.133)	0.009 (0.932)	0.070 (0.576)	0.131 (0.236)	0.104 (0.348)
Flow index	0.238 (0.026)*	0.285 (0.009)*	-0.062 (0.575)	-0.097 (0.382)	-0.083 (0.443)	0.205 (0.061)	-0.072 (0.517)	0.020 (0.857)	0.173 (0.117)

*WBC* White blood cell; *ANC* Absolute neutrophil count; *AMC* Absolute monocyte count; *ALC* Absolute lymphocyte count; *Plt* Platelet; *NLR* Neutrophil lymphocyte ratio; *LMR* Lymphocyte monocyte ratio; *PLR* Platelet lymphocyte ratio; *SII* Systemic inflammatory index

*significance for* p *< 0.05

## Discussion

Inflammation has long been implicated in the pathophysiology of AVMs, contributing to vessel wall destabilization through angiogenesis, extracellular matrix degradation, and cellular apoptosis [18, 38]. These processes are driven by genetic and hemodynamic factors and mediated by cytokines, neutrophils, and macrophages. However, the precise mechanisms by which inflammation influences AVM behavior remain incompletely understood. Several genetic polymorphisms have been identified as potent regulators of angiogenic and proinflammatory protein expression, thereby exacerbating AVM instability and increasing the risk of rupture [5, 6, 26]. Systemic inflammatory markers, such as the SII, have shown prognostic utility in various cerebrovascular disorders, including AVMs, but their relevance to AVM angioarchitecture and hemodynamic features remains uncertain [4, 20, 30, 31].

This study sought to evaluate associations between systemic inflammatory markers and specific features of AVMs, including size, volume, nidal compactness, venous stenosis, Spetzler-Martin grade, feeder artery aneurysms, intranidal aneurysms, and AVM flow. Our results did not reveal consistent or significant correlations between these markers and AVM characteristics. These findings contrast with those of Kurisu et al. (2024), who found elevated SII levels to be associated with increased severity of dural arteriovenous fistulas (dAVFs), linking SII to adverse features such as venous occlusion and pseudophlebitic patterns. [8] Moreover, in our previous study on surgically resected AVM tissue, CD68 staining was used to quantify macrophage infiltration within vessel walls [12]. This analysis revealed a negative association between macrophage infiltration and venous anomaly and a positive association with hemosiderin grade, which is linked to dense fibrosis and a potential predisposition to vessel leakage [21]. These contrasting findings highlight a possible disconnect between systemic inflammatory markers, like the SII, and the localized pathological processes within AVMs. This underscores the need for further exploration of alternative approaches to assess inflammation at the lesion level.

Histological studies offer insights that may explain these findings. Wright et al. (2020) demonstrated heavy macrophage infiltration within AVM walls, regardless of rupture status, indicating a baseline inflammatory state across all AVMs. [15] This uniform inflammation may dilute the discriminatory power and utility of systemic inflammatory markers like SII, which measure broader immune activity rather than lesion-specific changes. Furthermore, proteins implicated in angiogenesis and inflammation, such as vascular endothelial growth factor (VEGF), matrix metalloproteinases (MMPs), and interleukin-6 (IL-6), are overexpressed in AVMs but have yet to demonstrate reliable clinical utility [36, 37]. These findings underscore the complexity of AVM-associated inflammation and the importance of exploring localized biomarkers through advanced imaging or tissue analyses.

Although the clinical utility of systemic inflammatory markers in AVMs remains elusive, their potential role in stratifying patients by risk or guiding therapeutic interventions remains a critical area of research. For instance, early identification of inflammatory markers might pave the way for targeted anti-inflammatory therapies to modulate disease progression or enhance post-treatment outcomes [22]. Translating these insights into clinical practice, however, requires robust evidence linking inflammatory pathways to specific AVM features. Additionally, while this study did not directly examine the role of inflammation in AVM rupture, this remains a critical topic for future research, with potential implications for improving patient management and prognostication.

In conclusion, our study did not identify significant associations between inflammatory markers and angioarchitectural or hemodynamic features of unruptured sporadic AVMs. These findings highlight the complexity of AVM pathology and underscore the need for further investigation into the inflammatory pathways underlying AVM behavior. Future research should aim to integrate systemic and localized assessments of inflammation to better understand its role in AVM development, progression, and response to therapy, particularly in light of evidence that AVM hemodynamics change during treatment [25], ultimately advancing clinical management and therapeutic strategies.

## Limitations

This study has several limitations. As an observational, retrospective, single-center study, the results may not be generalizable to broader populations. It is important to note that our dataset predominantly comprises sporadic AVMs, as patients with syndromic or hereditary conditions (e.g., HHT or capillary malformation-AVM syndrome) were underrepresented. This limitation should be considered when interpreting our findings, and future research specifically targeting these subgroups is warranted. The inclusion criteria, which required complete baseline CBC data, may introduce a selection bias, as not all patients in our database had complete lab results. Further, the reliance on a single CBC measurement does not account for inherent fluctuations in inflammatory markers due to measurement variability and timing relative to patient presentation. Additionally, while patients with active infections were excluded to reduce inflammatory confounding, comprehensive data on other chronic inflammatory comorbidities (e.g., rheumatoid arthritis, chronic respiratory diseases) and medications that could affect WBC counts (such as steroids) were not fully available. This represents an important limitation of our study. Future studies should consider incorporating serial or averaged CBC measurements to enhance accuracy and robustness. Future multicenter, longitudinal studies incorporating both systemic and localized markers, as well as histological correlates, are necessary to validate and expand upon these findings.

## Data Availability

No datasets were generated or analysed during the current study.
